# Retinal microvascular signs and risk of diabetic kidney disease in asian and white populations

**DOI:** 10.1038/s41598-021-84464-7

**Published:** 2021-03-01

**Authors:** Simon Nusinovici, Charumathi Sabanayagam, Kristine E. Lee, Liang Zhang, Carol Y. Cheung, E. Shyong Tai, Gavin S. W. Tan, Ching Yu Cheng, Barbara E. K. Klein, Tien Yin Wong

**Affiliations:** 1 Singapore Eye Research Institute, Singapore National Eye Centre, 11 Third Hospital Avenue, Singapore, 168751 Singapore; 2 Ophthalmology and Visual Sciences Academic Clinical Programme, Duke-NUS Medical School, National University of Singapore, Singapore, Singapore; 3 Department of Ophthalmology and Visual Sciences, University of Wisconsin Medical School, Madison, WI USA; 4 Department of Biostatistics and Medical Informatics, University of Wisconsin Medical School, Madison, WI USA; 5 Department of Ophthalmology and Visual Sciences, The Chinese University of Hong Kong, Hong Kong, China; 6 Department of Medicine, National University Health System, National University of Singapore, Singapore, Singapore

**Keywords:** Epidemiology, Biomarkers, Diseases, Nephrology

## Abstract

The objective was to examine prospectively the association between retinal microvascular signs and development of diabetic kidney disease (DKD) in Asian and White populations. We analysed two population-based cohorts, composing of 1,221 Asians (SEED) and 703 White (WESDR) adults with diabetes. Retinal microvascular signs at baseline included vascular caliber (arteriolar—CRAE, and venular—CRVE) and diabetic retinopathy (DR). Incident cases of DKD were identified after ~ 6-year. Incident cases were defined based on eGFR in SEED and proteinuria or history of renal dialysis in WESDR. The incidence of DKD were 11.8% in SEED and 14.0% in WESDR. Wider CRAE in SEED (OR = 1.58 [1.02, 2.45]) and wider CRVE (OR = 1.69 [1.02, 2.80)) in WESDR were associated with increased risk of DKD. Presence of DR was associated with an increased risk of DKD in both cohorts (SEED: OR = 1.91 [1.21, 3.01] in SEED, WESDR: OR = 1.99 [1.18, 3.35]). Adding DR and retinal vascular calibers in the model beyond traditional risk factors led to an improvement of predictive performance of DKD risk between 1.1 and 2.4%; and improved classification (NRI 3 between 9%). Microvascular changes in the retina are longitudinally associated with risk of DKD.

## Introduction

Diabetic kidney disease (DKD) is a common and serious complication of diabetes. Around 40% of persons with diabetes develop DKD^1^. DKD is the leading cause of end-stage renal disease^2^ and is strongly associated with cardiovascular outcomes^3, 4^ and death^5^. Despite clear screening recommendations in people with diabetes (annual assessment once diabetes is diagnosed for type 2 diabetes)^6^, DKD remains substantially underdiagnosed^7–9^, which leads to lost opportunities for prevention and may contribute to disease progression. Early detection of patients at high risk for DKD may allow potential interventions to slow the progression of DKD to end-stage kidney disease, and avoid unnecessary treatment and costs in low risk patients^10^.

The eye shares structural, physiological and pathogenic pathways with the kidney^11^. The similarities between these two organs make them both susceptible to risk factors such as diabetes. Diabetes leads to functional and structural alterations in the microvasculature in the kidney and retina. Consequently, the two most common microvascular complications are DKD and diabetic retinopathy (DR), with clear parallels in their pathophysiology^11^. Several studies have investigated the association between a range of retinal microvascular signs, including changes in retinal arteriolar and venular calibers, and retinopathy (e.g., microaneurysms, retinal haemorrhages) with the risk of DKD^12–19^. However, most studies were conducted in cross-sectional settings^12–14, 16–19^, with only few prospective studies^20–24^. Moreover, while the relationship between DR and the increased risk of DKD is well established^12–19^, findings regarding the relationships between retinal vascular caliber and the risk of DKD remain inconclusive^20–24^. Furthermore, despite wide variations of DKD prevalence between ethnicities and countries^25, 26^, whether the relationship of retinal microvascular signs to risk of DKD in Asians is similar to Whites has not been investigated. Finally, to our knowledge, no study has investigated the incremental value of retinal vascular changes, in terms of discrimination and reclassification in DKD risk beyond traditional risk factors. An improvement in the prediction could help better identify individuals at risk of DKD and for subsequent management.

To address these gaps, we investigated the prospective association between retinal microvascular signs, including retinal vascular calibre and DR, and the risk of DKD in Asian and White populations, and determined the predictive values of these retinal signs in identifying patients at risk of developing DKD beyond traditional risk factors.

## Results

### Descriptive statistics

The two populations were composed of 1,221 participants in SEED and 703 participants in WESDR. The mean ages were 58.1 and 63.6, respectively. The incidence of DKD at the follow-up examination were 11.8% in SEED and 14.0% in WESDR. In the latter cohort, out of the 96 incident cases, only one went for dialysis. The prevalence of DR at baseline were 23.5% in SEED and 50.0% in WESDR, respectively (Table 1).

**Table 1:** Baseline characteristics of SEED and WESDR participants.

Characteristics	SEED N = 1221	WESDR N = 703
Incident DKD, n (%)	144 (11.8)	96 (14)
Any Diabetic retinopathy, n (%)	287 (23.5)	349 (50)
Retinal arteriolar calibre (CRAE, µm), mean (SD)	143 (14.8)	155.4 (14.18)
Retinal venular calibre (CRVE, µm), mean (SD)	212.2 (21.4)	236.2 (24.48)
Gender (male), n (%)	617 (50.5)	311 (44)
Age (years), mean (SD)	58.1 (8.6)	63.6 (10.86)
**Ethnicity, n (%)**		
Chinese	306 (25.1)	NA
Indian	570 (46.7)	NA
Malay	345 (28.3)	NA
Education level, n (%)*		
Category 1	721 (59)	296 (42)
Category 2	430 (35.2)	406 (58)
Category 3	70 (5.7)	–
Body Mass Index (kg/m^2^), mean (SD)	27 (4.7)	29.5 (5.85)
**Smoking status, n (%)**		
Never smoked	870 (71.3)	384 (55)
Current smoker	172 (14.1)	101 (14)
Past smoker	179 (14.7)	218 (31)
Systolic blood pressure (mmHg), mean (SD)	140.9 (19.4)	143.9 (20.96)
Blood glycosylated haemoglobin (%), mean (SD)	7.7 (1.6)^&^	9.5 (1.96)^$^
Duration of diabetes (years), mean (SD)	5.8 (7.5)	10.3 (7.45)
Baseline level of eGFR (mL/min/1.73m^2^), mean (SD)	89.8 (14.1)	NA
Hypertensive treatment, n (%)	582 (47.7)	322 (46)
History of cardiovascular disease, n (%)	161 (13.2)	141 (20)
Total Cholesterol (mmol/L), mean (SD)	5.1 (1.1)	NA

*WESDR: category 1 = grade 1 up to diploma, category 2 = degree and above; SEED: category 1 = no formal education or primary education only, category 2 = O/N/A levels, category 3 = university education.

^&^HbA1c; ^$^ total glycosylated haemoglobin.

### Association between retinal vascular abnormalities and DKD

Firstly, the associations between retinal vascular abnormalities (RVA) (CRAE, CRVE and presence of DR) and DKD were investigated, independently of traditional risk factors (Table 2). The presence of DR was associated with an increased risk of DKD in both cohorts (OR = 1.91 [1.21, 3.01] in SEED and OR = 1.99 [1.18, 3.35] in WESDR, respectively). In SEED, wider CRAE was associated with an increased risk of DKD (OR = 1.58 [1.02, 2.45]), while in WESDR, wider CRVE was associated with an increased risk of DKD (OR = 1.69 [1.02, 2.80]) (Table 2).

**Table 2:** Associations between vascular calibers and the incidence of diabetic kidney disease (DKD) according to the DR status using multivariable logistic model in SEED and WESDR cohorts.

	SEED (n = 1221)			WESDR (n = 703)		
	DKD incidence	OR + 95%CI	*p*-value	DKD incidence	OR + 95%CI	*p*-value
**CRAE**						
Narrow	10.8	1		13.82	1	
Wide	12.8	1.58 [1.02, 2.45]	0.04	13.5	0.81 [0.49, 1.34]	0.42
**CRVE**						
Narrow	10.5	1		10.88	1	
Wide	13.1	1.15 [0.73, 1.8)	0.55	16.25	1.69 [1.02, 2.80)	0.04
**DR**						
No sign	8.9	1		8.47	1	
Any lesion	21.3	1.91 [1.21, 3.01]	0.006	18.91	1.99 [1.18, 3.35]	0.01

Central retinal arteriolar equivalent (CRAE) and central retinal venular equivalent (CRVE) were considered narrow if ≤ median value and wide if > median value. The models were adjusted for age, gender, education, BMI, smoking status, systolic blood pressure, blood glycosylated haemoglobin, duration of diabetes, hypertensive treatment and history of cardiovascular events (with further adjustment on ethnicity, baseline level of eGFR and total cholesterol in SEED).

### DKD prediction using retinal vascular abnormalities

**Figure 1:**
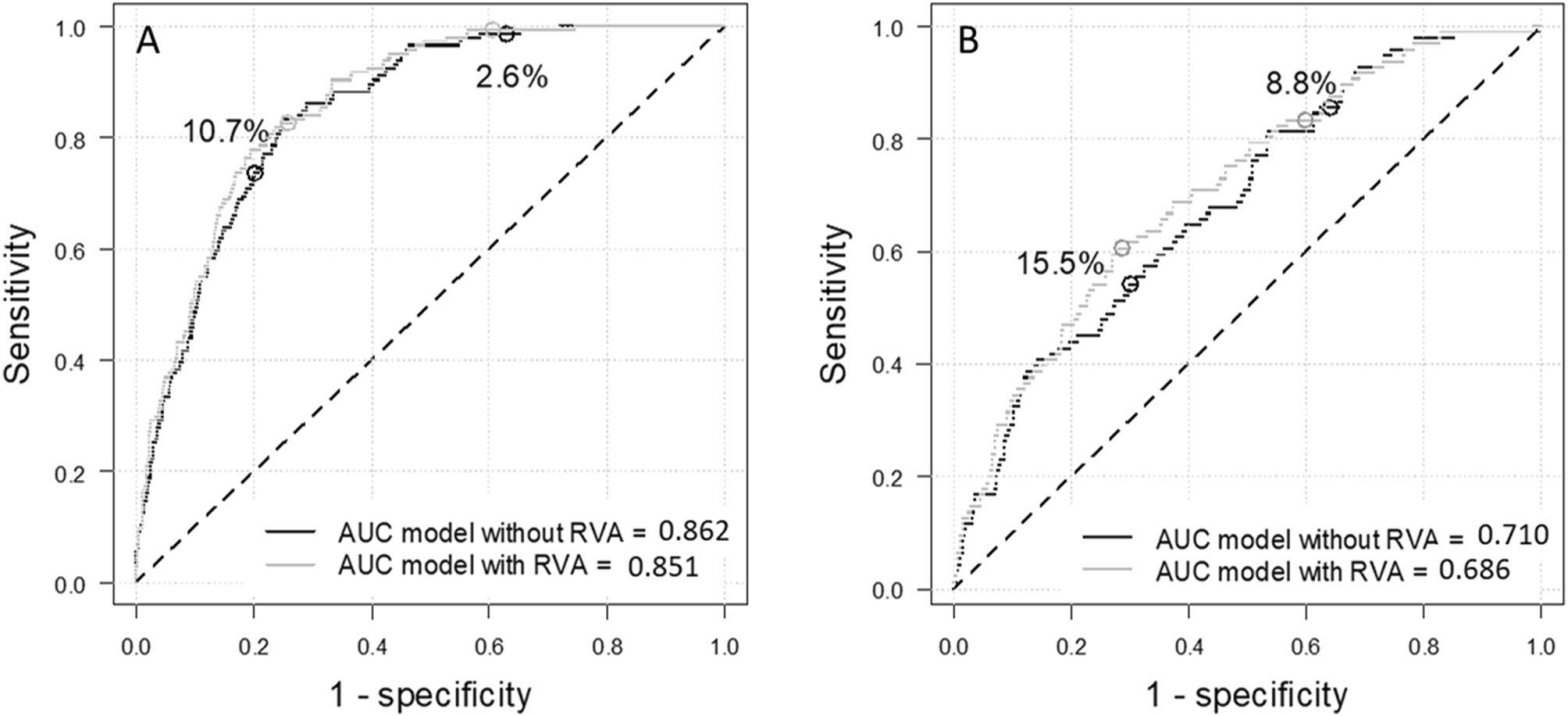
Area under the ROC curve for models with and without retinal vascular abnormalities (RVA) in (A) SEED (n = 1221) and (B) WESDR cohorts (n = 703). The risk thresholds for diabetic kidney disease (DKD) prediction correspond to the tertiles of predicted values in the model without RVA (SEED: 2.6 and 10.7%; WESDR: 8.8 and 15.5%).

Adding RVA in the model beyond traditional risk factors led to a significant improvement of the area under the ROC curve only in SEED cohort (SEED: 0.862 versus 0.851, *p* = 0.01; WESDR: 0.710 versus 0.686 *p* = 0.10) (Fig. 1). Moreover, in SEED, only the addition of both DR and vessel calibers increase the predictive performance (DR or vessel calibers alone were non-significant, Supplementary Materials Table 1). Adding RVA allowed to better classify participants without DKD in both cohorts (SEED: NRI = 3.25%; WESDR: 5.44%) and participants with DKD only in WESDR cohort (NRI = 3.12%) (Table 3).

**Table 3:** Reclassification of diabetic kidney disease (DKD) risk after addition of retinal vascular abnormalities (RVA) in the prediction model in SEED (n = 1221) and WESDR (n = 703).

SEED (n = 1221)	Participants with DKD					Reclassified as higher risk	Reclassified as lower risk	NRI (%)
	Model with RVA					5	5	0
		Low	Interm	High	Total			
Model without RVA	Low	1	1	0	2			
	Interm	0	18	4	22			
	High	0	5	115	120			
	Total	1	24	119	144			

SEED (n = 1221)	Participants without DKD					Reclassified as higher risk	Reclassified as lower risk	NRI (%)
	Model with RVA					67	102	3.25
		Low	Interm	High	Total			
Model without RVA	Low	375	30	0	405			
	Interm	53	294	37	384			
	High	0	49	239	288			
	Total	428	373	276	1077			
							NRI total (%)	3.25

WESDR (n = 703)								
	Participants with DKD					Reclassified as higher risk	Reclassified as lower risk	NRI (%)
	Model with RVA					16	13	3.12
		Low	Interm	High	Total			
Model without RVA	Low	7	6	1	14			
	Interm	9	12	9	30			
	High	0	4	48	52			
	Total	16	22	58	96			

WESDR (n = 703)	Participants without DKD					Reclassified as higher risk	Reclassified as lower risk	NRI (%)
	Model with RVA					85	118	5.44
		Low	Interm	High	Total			
Model without RVA	Low	182	35	3	220			
	Interm	60	98	47	205			
	High	2	56	124	182			
	Total	244	189	174	607			
							NRI total (%) 8.56	

The risk thresholds for DKD prediction correspond to the tertiles of predicted values in the model without RVA (SEED: low: 0–2.6%; Intermediate: 2.6–10.7%; and high: > 10.7%; WESDR: low: 0–8.8%; Intermediate: 8.8–15.5%; and high: > 15.5%).

## Discussion

In our study, we first showed that retinal microvascular signs, including DR and wider retinal vessel calibres were associated with the incidence of DKD in Asian and White cohorts. Our study is the first to prospectively evaluate these relationships comparing two population-based cohorts in Whites and Asian populations. Second, we showed that adding RVA beyond traditional risk factors increased the predictive performance with up to 9% of the individuals being reclassified.

Differences between the two cohorts should be discussed. First, we used two incident case definitions, one based mainly on proteinuria (WESDR) and one based on eGFR (SEED). Although proteinuria appears early in DKD, significant proteinuria can occur in advanced cases, we cannot therefore conclude about potential differences in DKD stages between the two populations. However, the incidence were very similar, between 12 and 14%. Second, the study periods were different, with older data collection for WESDR (1980–1986) compared to SEED (2004–2011). That could explain the difference in DR prevalence, with DR being almost two times more frequent in WESDR cohort. This difference might be due to difference in treatment options available for DR during these two time periods. However, despite these differences, the magnitude of effects of the associations between DR and DKD were very similar in the two cohorts.

We found that in the white population, wider venular calibers were associated with an increased risk of DKD. This result is in accordance with previous studies^19–21^ (Supplementary Materials Table 2). Wider venular caliber is related to systematic markers of inflammation^27–29^. However, McKay et al., 2018 did not find any association between venular caliber and variations of eGFR. As the authors stated in their discussion, the 3-year duration between baseline and follow-up measures might have been insufficient to detect associations between retinal vessel calibers and variations of eGFR. In the Asian population, on the contrary, wider arteriolar calibers were associated with an increased risk of DKD. These results between the two cohorts could suggest different pathophysiology in different DKD stages. None of the previous studies conducted in Asian populations has reported an association between wider arteriolar calibers and the risk of DKD in the multivariable models^22, 23^. This absence of effect is possibly due to the small sample sizes. In Yip et al., 2015, the outcome considered was end-stage renal failure with only 33 incident cases^22^ and in Yip et al., 2017, among 78 participants that developed CKD, 55 had diabetes^23^. Overall, the results are biologically meaningful because both venular and arteriolar widening have been reported in diabetic populations^30^. It is likely that both dilation in retinal vessels and DR are reflective of cumulative microvascular damage which eventually results in DKD. These microvascular damages could result from different mechanisms including inflammation and oxidative stress. Inflammation that is associated with wider retinal venular caliber^27–29^, also contributes to the development and progression of DKD, through the increased expression of inflammatory-associated mediators^31^. The second mechanism involves advanced glycation end products (AGEs) synthesized during high oxidative stress or hyperglycemia. AGEs can lead to cellular hypertrophy and apoptosis in the kidney^32^, and to calcification and apoptosis in the eye^33, 34^. High serum AGE levels, as found in patients with chronic kidney disease, have been shown to trigger retinopathy to a comparable extent as that seen in people with diabetes^35, 36^. The vascular damages occurring in the eyes could therefore reflect at early stages vascular damages in the kidney.

Retinal photographs are regularly captured for DR screening in patients with diabetes. The retinal photography and the prediction model with retinal microvascular signs offer an opportunistic screening of DKD. It has been shown that retinal vascular calibers can help better predict DR^37^ and stroke events^38^, beyond established risk factors. In this study, retinal vascular abnormalities allowed to reclassify up to 9% of individuals in a more appropriate risk category (higher risk category for cases and lower category for controls). This effect was more pronounced for the reclassification of individuals without DKD, corresponding to an increase in specificity. In terms of discrimination, we showed only small improvement in the AUC in the Asian population (equal to 1.1%). However, It has indeed been demonstrated that enormous odds-ratios are required to meaningfully increase the AUC^39, 40^ and a 0.01 increase of AUC might suggest a meaningful improvement^41^. In the US population, the increase in discrimination was higher in terms of magnitude of effect (equal to 2.4%) but statistically non-significant.

In people with type 2 diabetes, the recommendations stipulate that both albuminuria and serum creatinine levels should be assessed annually for DKD once diabetes is diagnosed. Likewise, annual screening for retinopathy is also recommended in diabetic patients. Retinal vascular assessment could thus be used in the screening process to help stratify patients for their risk of developing DKD. This could allow identifying earlier patients at risk for DKD for whom treatment can slow the progression of DKD. More studies are needed to confirm these results. Moreover, the clinical usefulness (utility and usability) and the cost-effectiveness have to be evaluated to account for both benefits and costs associated with the utilization of fundus photographs.

Strengths of our study include the longitudinal design and the inclusion of two prospective population-based cohorts from Asia and from the US. This study have, however, some limitations. Firstly, the sample size was limited for the cohort in the US population, we cannot thus conclude whether there was no improvement in discrimination or whether the statistical power was too limited. Secondly, the two studies were not collected at the same time period. Thirdly, although proliferative diabetic retinopathy (PDR) has been shown to be a highly specific indicator for diagnosing diabetic nephropathy, we could not assess its effect on DKD due to very limited numbers (in SEED, there were only 4 DKD events among 15 participants with PDR). Fourthly, the findings in WESDR study may be influenced by the method used to measure proteinuria. Factors that affect renal hemodynamics such as medications, state of hydration and dietary protein intake may lead to false-positive dipstick test results. Moreover, using a single measurement may lead to an overestimation of the incidence compared with the use of multiple measurements over time. Finally, none of the population had both available proteinuria and serum creatinine. Further longitudinal studies with both parameters are needed to further investigate the ability of retinal vasculature to predict early and advanced DKD cases.

Our study confirms the existence of longitudinal associations between signs of RVA and the risk of DKD. RVA improve the discrimination and classification of DKD cases beyond traditional risk factors, which highlight the potential usefulness of retinal vasculature in patient stratification.

## Materials and methods

We conducted the analyses based on two prospective cohort studies, the Singapore Epidemiology of Eye Disease (SEED) and the Wisconsin Epidemiologic Study of Diabetic Retinopathy (WESDR).

### Study populations

SEED is a population-based study of eye diseases in Asian Chinese, Indian and Malay adults aged 40–80 years. Details of the study participants and methods have been reported elsewhere^42, 43^. Participants underwent a standardised interview, ocular examination and laboratory investigations at both baseline and follow-up visits. The average length of follow-up between the two visits was 6 years. Informed, written consent was obtained from participants, and ethical approval was obtained from the Institutional Review Board of the Singapore Eye Research Institute. Furthermore, all research was performed in accordance with relevant guidelines and regulations. Out of the participants examined at baseline and follow-up visits, 1,770 had diabetes. Since this study aims at investigating risk factors of incident DKD, participants with DKD at baseline were excluded (n = 265 prevalent DKD). We further excluded participants with missing eGFR at baseline (n = 50) and follow-up visit (n = 90), and incident cases with less than 25% decrease in estimated glomerular filtration rate (eGFR) between baseline and follow-up examinations (n = 49). Finally, we excluded participants with incomplete medical records (n = 95). The missing information concerned: DR status (n = 21), retinal vascular parameter (n = 64), education level (n = 3), body mass index (BMI) missing (n = 2), smoking status (n = 1), level of glycosylated A1c haemoglobin (HbA1c) (n = 9), hypertensive medication (n = 14) and cardiovascular diseases (n = 3). The population included in the analyses was composed of 1,221 participants.

WESDR is a population-based study of eye diseases conducted in Wisconsin in people with diabetes of all ages. Details of the study participants and methods have been reported elsewhere^44^. The baseline examination was conducted on a total of 1,370 participants who were diagnosed as having diabetes at 30 years of age or older. The average length of follow-up between the two visits was 5 years. People with DKD at baseline were excluded (196 prevalent DKD + 38 missing DKD information). Out of these people, 297 without follow-up visit and 45 without information on DKD at the follow-up visit were excluded. We finally excluded participants with missing information on retinal vascular parameter (n = 26) or one of the covariates (n = 65). The missing information for the covariates concerned: total glycated hemoglobin (total GHb) (n = 62), anti-hypertensive medication (n = 1), history of cardiovascular disease (n = 2) and systolic blood pressure (n = 1). The population included in the analyses was composed of 703 participants.

### Definition of incident DKD

Incident cases of DKD were identified at the follow-up visit (among participants free of DKD at baseline) based on onset of DKD defined as: In SEED, DKD was defined as estimated glomerular filtration rate (eGFR) < 60 mL/min/1.73m^2^ accompanied by ≥ 25% decrease in eGFR from the baseline. eGFR was estimated from serum creatinine using the chronic kidney disease epidemiology collaboration (CKD-EPI) equation to account for variations in creatinine levels due to age, gender and body weight^45^. In WESDR, DKD was defined as presence of proteinuria (protein concentration of ≥ 0.30 g/L as measured by a urine dipstick—Labstix; Ames, Elkhart, IN) or history of kidney transplant or history of renal dialysis^20^.

### Assessment of retinal microvascular signs

We evaluated two retinal microvascular signs: retinal vascular caliber measurements and presence of DR. In both cohorts, colour retinal photographs of both eyes were taken after dilating the pupils. The image with the best quality for one eye was graded for retinal vascular calibre measurements by using computer-assisted software (IVAN; University of Wisconsin, Madison, Wisconsin)^46^ by a trained grader who was masked to participants characteristics. All arterioles and venules coursing through a specific area from 0.5- to 1-disc diameter from the optic disc margin were measured and combined into summary measures, referred to as “central retinal arteriolar equivalent” (CRAE) and “central retinal venular equivalent” (CRVE). The reproducibility of retinal vascular measurements was found to be very high, with intragrader intraclass correlation coefficients of 0.99 (95% confidence interval (CI) 0.98, 0.99) for CRAE and 0.94 (95% CI 0.92, 0.96) for CRVE^47^.

DR was considered present if any characteristics lesions as defined by the Early Treatment for Diabetic Retinopathy Study (ETDRS) severity scale was present: microaneurysms, hemorrhages, cotton wool spots, intraretinal microvascular abnormalities, hard exudates, venous beading and neovascularization^48^.

### Traditional risk factors of DKD

In both cohorts, the following risk factors were considered: age, gender, educational level (primary education or lower, secondary and postsecondary), body mass index (BMI), smoking status (never, current and past smoker), systolic blood pressure, blood glycated haemoglobin (total GHb in WESDR and HbA1c in SEED), duration of diabetes, hypertensive medication (yes versus no) and self-reported history of cardiovascular disease (yes versus no). Only covariates that showed a linear relationship (checked using generalized additive models) with the risk of DKD were considered as continuous in the model, i.e., age, systolic blood pressure, blood glycated haemoglobin and baseline level of eGFR (only in SEED). The other continuous covariates were considered as categorical: BMI (BMI < 25, BMI 25–30 and BMI > 30 kg/m^2^), duration of diabetes (< 1, 1–10 and ≥ 10 years) and total cholesterol (< 4, 4–6 and > 6 mmol/L). The cut-off values were based on visual inspection of the smoothed functions and on clinical expertise. In SEED cohort, ethnicity (Chinese, Indian and Malay) and total cholesterol were also considered.

### Statistical analyses

Multivariable logistic models were built to investigate the association between the three measures of RVA (i.e. CRAE, CRVE and presence of DR), and the incidence of DKD, independently of the traditional risk factors. CRAE and CRVE were considered either as continuous or binary. In the latter case, narrower and wider CRAE (or CRVE) were considered for calibers lower and higher than the median population value, respectively.

To investigate the improvement in DKD risk prediction by using RVA beyond traditional risk factors, the change in discrimination and the reclassification were calculated between the models with and without RVA. The discrimination measures how well a prognostic model distinguishes individuals with and without the outcome of interest. The reclassification quantifies how much a new prognostic marker increases the proportion of individuals correctly re-classified as having or not having a given event compared to a previous classification based on an existing predictive model. To evaluate the change in discrimination, we calculated the improvement in the area under the ROC curve for the model with RVA compared to the model without (ΔAUC).

The reclassification was evaluated using the Net Reclassification Index (NRI), reported separately for events and non-events. The calculation of NRI requires cut off to define which individuals should be considered at low or high risk (or at low, intermediate and high risk if three categories are considered). To the best of our knowledge, no standard risk threshold exist for DKD incidence prediction. Therefore, two cut off values were used corresponding to the tertiles of predicted values in the model without RVA (the same cut off points were considered for both models). These values corresponded to predicted probabilities of being DKD. These cut off points were used to classify participants to low, intermediate or high risk categories. The analyses were done using R software (version 3.4.4) and SAS (version 9.4r).

## Supplementary Information

Supplementary information.

## Data Availability

In order to adhere to local IRB guidelines, we regret to inform that we are unable to share data relevant to this study openly. Nevertheless, we respectfully urge interested parties to contact corresponding author for further enquiries if necessary.
