# Supplementary material for: Retinal microvascular signs and risk of diabetic kidney disease in asian and white populations
Source: Sci Rep. 2021 Mar 1;11:4898. doi: 10.1038/s41598-021-84464-7 (PMC7921402; doi:10.1038/s41598-021-84464-7)
Supplement: Supplementary file 1 — Supplementary information. [file 41598_2021_84464_MOESM1_ESM.pdf]

<u>Supplementary Materials</u>: Retinal Microvascular Signs and Risk of Diabetic Kidney Disease in Asian and White populations

Simon Nusinovici PhD[1], Charumathi Sabanayagam MD, PhD[1,2], Kristine E Lee MS[3,4], Zhang Liang PhD[1], Carol Y Cheung PhD[1,5], E Shyong Tai MD, PhD[6], Gavin SW Tan MD[1,2], Ching Yu Cheng MD, PhD[1], Barbara E.K. Klein MD, PhD[3], Tien Yin Wong MD, PhD[1,2*]

[1]Singapore Eye Research Institute, Singapore National Eye Centre, Singapore, Singapore

[2]Ophthalmology and Visual Sciences Academic Clinical Programme, Duke-NUS Medical School, National University of Singapore, Singapore

[3]Department of Ophthalmology and Visual Sciences, University of Wisconsin Medical School, Madison, Wisconsin

[4]Department of Biostatistics and Medical Informatics, University of Wisconsin Medical School, Madison, Wisconsin

[5]Department of Ophthalmology and Visual Sciences, the Chinese University of Hong Kong, Hong Kong

[6]Department of Medicine, National University Health System, National University of Singapore, Singapore

*Corresponding author

Prof. Tien Y Wong, Singapore National Eye Centre, 11 Third Hospital Avenue, Singapore 168751, T: +65 6322 8333 / F: +65 62263395 / M: +65 98565354 / Email: wong.tien.yin@singhealth.com.sg

Table 1. Area under the ROC curve for models with and without retinal vascular abnormalities (RVA) in (A) SEED (n=1,221) and (B) WESDR cohorts (n=703). Four models were considered: (1) traditional risk factors only, (2) traditional risk factors with vessel calibers (CRAE and CRVE), (3) traditional risk factors with signs of diabetic retinopathy, or (4) traditional risk factors with vessel calibers and signs of diabetic retinopathy.

	SEED		WESDR	
	AUC	p-value[$]	AUC	p-value[$]
Tradition risk factors only (1)	0.851 [0.82, 0.88]	NA	0.686 [0.631, 0.742]	NA
Tradition risk factors + vessel calibers (2)	0.855 [0.83, 0.88]	0.13	0.697 [0.642,0.752]	0.27
Tradition risk factors + signs of diabetic retinopathy (3)	0.857 [0.83, 0.88]	0.11	0.701 [0.646, 0.756]	0.23
Tradition risk factors + vessel calibers and signs of diabetic retinopathy (4)	0.862 [0.84, 0.89]	0.02	0.71 [0.656, 0.765]	0.10

[$] p-values corresponded to the comparison of the model with traditional risk factors alone and one of the other model further including vessel calibers (2), signs of diabetic retinopathy (3) or vessel calibers and signs of diabetic retinopathy (4)

NA: not applicable

Table 2. Population-based prospective studies conducted in adults with diabetes investigating the associations between retinal microvascular signs and risk of diabetic kidney disease

Author, year	Type of study, location	Population, follow-up	Retinal microvascular signs	DKD definition	Results
Wong et al., 2004	Population-based cohort study (WESDR), US	People with type 1 diabetes diagnosed before 30 years without existing gross proteinuria or renal insufficiency at baseline (n=557), 16-year follow-up	CRAE, CRVE	Incidence of gross proteinuria (urine protein ≥0.30g/L) and renal insufficiency (eGFR <60 mL/min/1.73 m^2)	Larger retinal venular diameter associated with increased risk of gross proteinuria (RR = 1.53 [1.19, 1.97] comparing quartile 4 versus 1-3) and with renal insufficiency (RR = 1.53 [1.19, 1.97]). No association with retinal arteriolar diameter
Klein et al., 2007	Population-based cohort study (WESDR), US	Adults diagnosed with diabetes at ≥30 years without existing gross proteinuria at baseline (n=533), 14-year follow-up	CRAE, CRVE	Incidence of gross proteinuria (urine protein ≥0.30g/L), starting renal dialysis, or undergoing a renal transplant	Larger CRVE associated with the 14-year incidence of diabetic nephropathy (OR fourth vs. first to third quartiles = 2.08; 95% CI, 1.47-2.94; P<0.001). No association with retinal arteriolar diameter
Yip et al., 2015	Population-based cohort study (SiMES, SP2), Singapore	Adults aged ≥40 years without baseline ESRD (n=5,763)	Retinopathy, CRAE, CRVE, fractal dimensions	ESRD (defined as eGFR <15 mL/min/1.73 m^2, serum creatinine ≥5.7mg/dl, or need for treatment for kidney failure)	Overall, retinopathy associated with incident ESRD (HR = 2.51 [1.14, 5.54]). In people with diabetes, retinopathy also associated with incident ESRD (HR = 2.60, 95%CI: 1.01-6.66), not in people without diabetes. CRAE, CRVE and retinal vascular fractal dimension were

					not associated with ESRD (overall and subset analysis).
Yip et al., 2017	Retrospective population-based study (SiMES)	Malay adults aged 40-80 years without CKD at baseline (n=1,256)	CRAE, CRVE, tortuosity, fractal dimension, branching angle, retinopathy	Incident CKD (eGFR <60 mL/min/1.73 m^2 and ≥25% decrease during follow-up of ~6 years)	Overall, smaller retinal arterioles (HR = 1.34 [1.00, 1.78]), larger retinal venules (2.35 [1.12-5.94]) and presence of retinopathy (2.54 [1.48-4.36]) were associated with incident CKD. In people with diabetes, only presence of retinopathy was associated with incidence of CKD (HR = 3.78 [1.72, 8.32]).
McKay et al., 2018	Nested longitudinal case-control study (GoDARTS), Scotland	People with type 2 diabetes without DKD at baseline (n=1,072)	CRAE, CRVE, AVR, number of first vessel branches within a pre-defined zone C, fractal dimension and vessel tortuosity	Incident DKD progressors (eGFR <60 mL/min/1.73 m^2 or a reduction in eGFR of at least 15% between baseline and follow-up)	No associations between baseline retinal vascular parameters and risk of eGFR progression
